# Multisystem inflammatory syndrome in children (MIS-C): A nationwide collaborative study in the Greek population

**DOI:** 10.1007/s00431-023-05383-5

**Published:** 2024-01-12

**Authors:** Stavroula Lampidi, Despoina Maritsi, Marietta Charakida, Irini Eleftheriou, Evangelia Farmaki, Nikos Spyridis, Konstantina Charisi, Petrina Vantsi, Filippos Filippatos, Kleopatra Skourti, Efimia Papadopoulou-Alataki, Kyriaki Papadopoulou-Legbelou, Parthena Kampouridou, Ioanna N. Grivea, Eleni Vergadi, Despoina Gkentzi, Despina Dimou, Patra Koletsi, Lampros Fotis, Theodota Liakopoulou, Aikaterini Agrafiotou, Katerina Kourtesi, Georgios Tsolas, Dimitrios Kafetzis, Vassiliki Papaevangelou, Gabriel Dimitriou, Emmanouil Galanakis, George A. Syrogiannopoulos, Vassiliki Spoulou, Athanasios Michos, Emmanuel Roilides, Maria N. Tsolia

**Affiliations:** 1 Second Department of Paediatrics, National and Kapodistrian University of Athens, “P. and A. Kyriakou” Children’s Hospital, 11527 Athens, Greece; 2 First Department of Paediatrics, Aristotle University of Thessaloniki, Hippokration General Hospital, 54642 Thessaloniki, Greece; 3 Third Department of Paediatrics, Aristotle University of Thessaloniki, Hippokration General Hospital, 54642 Thessaloniki, Greece; 4 First Department of Paediatrics, National and Kapodistrian University of Athens, ‘Aghia Sophia’ Children’s Hospital, 11527 Athens, Greece; 5 Fourth Department of Pediatrics, Aristotle University of Thessaloniki, Papageorgiou General Hospital, 56429 Thessaloniki, Greece; 6 Pediatric Department, Genimatas General Hospital, 54635 Thessaloniki, Greece; 7 Department of Paediatrics, Faculty of Medicine, School of Health Sciences, University of Thessaly, 41500 Larissa, Greece; 8 Department of Paediatrics, Medical School, University of Crete, 71003 Heraklion, Greece; 9 Department of Paediatrics, University of Patras, University General Hospital of Patras, 26504 Patra, Greece; 10 Paediatric Department, Penteli Children’s Hospital, 15236 Athens, Greece; 11 Third Department of Paediatrics, National and Kapodistrian University of Athens, General University Hospital “Attikon”, 12462 Athens, Greece; 12 Department of Paediatrics, Metropolitan Hospital, 18547 Athens, Greece; 13 IASO Children’s Hospital, 15123 Athens, Greece

**Keywords:** Multisystem inflammatory syndrome in children, Paediatric inflammatory multisystem syndrome, MIS-C, PIM-TS, SARS-CoV-2, COVID-19

## Abstract

Multisystem inflammatory syndrome in children (MIS-C) is a rare but severe hyperinflammatory condition that may occur following SARS-CoV-2 infection. This retrospective, descriptive study of children hospitalized with multisystem inflammatory syndrome in children (MIS-C) in 12 tertiary care centers from 3/11/2020 to 12/31/2021. Demographics, clinical and laboratory characteristics, treatment and outcomes are described. Among 145 patients (95 males, median age 8.2 years) included, 123 met the WHO criteria for MIS-C, while 112 (77%) had serological evidence of SARS-CoV-2 infection. Fever was present in 99%, gastrointestinal symptoms in 77%, mucocutaneous involvement in 68% and respiratory symptoms in 28%. Fifty-five patients (38%) developed myocarditis, 29 (20%) pericarditis and 19 (13%) coronary aneurysms. Among the above cases 11/55 (20%), 1/29 (3.4%) and 5/19 (26.3%), respectively, cardiac complications had not fully resolved at discharge. Underlying comorbidities were reported in 18%. Median CRP value was 155 mg/l, ferritin 535 ng/ml, PCT 1.6 ng/ml and WBC 14.2 × 10^9^/mm^3^. Most patients had elevated troponin (41.3%) and/or NT-pro-BNP (49.6%). Intravenous immunoglobulin plus corticosteroids were used in 117/145 (80.6%), monotherapy with IVIG alone in 13/145 (8.9%) and with corticosteroids alone in 2/145 (1.3%). Anti-IL1 treatment was added in 15 patients (10.3%). Thirty-three patients (23%) were admitted to the PICU, 14% developed shock and 1 required ECMO. Mortality rate was 0.68%. The incidence of MIS-C was estimated at 0.69/1000 SARS-CoV-2 infections. Patients who presented with shock had higher levels of NT-pro-BNP compared to those who did not (*p* < 0.001). Acute kidney injury and/or myocarditis were associated with higher risk of developing shock.

*Conclusion*: MIS-C is a novel, infrequent but serious disease entity. Cardiac manifestations included myocarditis and pericarditis, which resolved in most patients before discharge. Timely initiation of immunomodulatory therapy was shown to be effective. NT-pro-BNP levels may provide a better prediction and monitoring of the disease course. Further research is required to elucidate the pathogenesis, risk factors and optimal management, and long-term outcomes of this clinical entity.

**Table:** 

**What is Known:** *• MIS-C is an infrequent but serious disease entity.* *• Patients with MIS-C present with multi-organ dysfunction, primarily involving the gastrointestinal and cardiovascular systems.*	
**What is New:** *• NT-pro-BNP levels may provide a better prediction and monitoring of the disease course.* *• Acute kidney injury and/or myocarditis were associated with higher risk of developing shock.*

**What is Known:**

*• MIS-C is an infrequent but serious disease entity.*

*• Patients with MIS-C present with multi-organ dysfunction, primarily involving the gastrointestinal and cardiovascular systems.*

**What is New:**

*• NT-pro-BNP levels may provide a better prediction and monitoring of the disease course.*

*• Acute kidney injury and/or myocarditis were associated with higher risk of developing shock.*

## Introduction

The COVID-19 pandemic, caused by the severe acute respiratory syndrome coronavirus 2 (SARS-CoV-2), has brought widespread changes in everyday life and routines among adults and children.

SARS-CoV-2 belongs to the coronavirus family, a subgroup of viruses which normally causes mild respiratory tract infections in humans and animals. Most people are infected at least once in their lives with coronaviruses, presenting with symptoms of common cold (fever, rhinorrhea, and sneezing). Rarely, coronaviruses mutate and may be transmitted from animals to humans, as happened, with the SARS (2003) and MERS (2012) epidemics, two viruses of zoonotic origin [1].

The new virus appeared in December 2019 in Wuhan, China. Since then, more than 600 million cases of SARS-CoV-2 infection have been reported worldwide. This highly transmissible virus spread rapidly in most countries, leading to the pandemic [1–3].

Most pediatric patients infected with SARS-CoV-2 were asymptomatic or presented with mild symptoms that did not require hospitalization or specific antiviral treatment [1, 4].

Nonetheless, a minority of children infected with SARS-CoV-2 develop a delayed immune-related response to the infection known as multisystem inflammatory syndrome in children (MIS-C) or pediatric inflammatory syndrome temporarily associated with SARS-CoV-2 (PIM-TS) [5]. PIM-TS was first described in April 2020 by the Royal College of Paediatrics and Child Health in the UK, where clinicians reported an unexplained cluster of patients presenting with hyperinflammatory shock [6]. This entity presented partially overlapping features with Kawasaki disease, toxic shock syndrome and/or macrophage activation syndrome, but could not be included under the same umbrella. In May 2020, the Centers for Disease Control and Prevention (CDC) and the World Health Organization (WHO) published their official case definitions of this new syndrome named MIS-C [7, 8].

Young patients with MIS-C appear unwell with fever and multi-organ dysfunction, primarily involving the gastrointestinal and cardiovascular system. In most cases, a mild or asymptomatic infection with COVID-19 4–6 weeks earlier is reported [9–13]. According to the case definition of this clinical entity, elevated inflammatory markers, lack of an alternative cause of inflammation and evidence of SARS-CoV-2 infection or exposure is required, to fulfill the diagnostic criteria.

The purpose of this study was to collect data on all children hospitalized with the diagnosis of MIS-C in Greece and to describe their demographic, clinical and laboratory features, the therapeutic decisions made, as well as their outcomes.

## Methods

### Study design

This was a national, multicenter, retrospective study of all patients hospitalized with MIS-C in 12 tertiary care centers in Greece from the beginning of the pandemic until December 31, 2021.

### Participants

This study was conducted by the Second Department of Paediatrics “P. and A. Kyriakou” Children’s Hospital, National and Kapodistrian University of Athens, in collaboration with all University Paediatric Departments throughout the country. The complete list of the Units and Hospitals involved may be found in the footnote.^1^

### Case ascertainment, data collection

Patients who met the case definition of MIS-C as proposed by the WHO or by the CDC were included. Patients who did not meet all the criteria, but their clinical presentation, resembled MIS-C were also included (MIS-C like) as possible cases, considering the case definition of the Royal College of Paediatrics and Child Health.

We collected data on demographics, clinical and laboratory characteristics, as well as comorbidities, treatment options and outcomes. We further stratified our cohort based on disease severity, by reporting on the percentage of patients requiring admission to the pediatric intensive care unit (PICU), as well as the need for mechanical ventilation, inotropic support or extracorporeal membrane oxygenation (ECMO).

## Results

A total of 145 patients recruited from 12 tertiary pediatric departments throughout the country, were enrolled in the study.

According to the case definition of the MIS-C by the WHO or CDC, 123 out of the 145 patients fulfilled all the criteria (85%). Seven (2%) patients did not present with multi-organ dysfunction, fourteen (10%) did not have evidence of prior SARS-CoV-2 infection or exposure and one (0.75%) patient did not have elevated inflammatory markers.

### Demographic characteristics

The median age of patients was 8.2 years (IQR 4.2–13.1), (range 3 months to 18 years). Only one infant was younger than 6 months of age. Male predominance (65.5%) was noted.

Twenty-six patients (18%) reported an underlying health condition (Table 1). The most common was asthma (5/145, 3.4%), followed by obesity (BMI > 95th percentile) (4/145, 2.7%) and type 1 diabetes mellitus (2/145, 1.3%).

**Table 1:** Underlying co-morbidities of reported cases

**Underlying comorbidities**		
	**Number**	**Percentage**
Asthma	5	3.4
Obesity	4	2.8
Diabetes mellitus	2	1.4
ASD, VSD	2	1.4
Heart valve disease	1	0.7
Beta-Thalassemia	1	0.7
Premature	2	1.4
Portal vein thrombosis	1	0.7
Urticaria	1	0.7
Periodic fever syndrome	1	0.7
Ozena	1	0.7
Lymphoma	1	0.7
Duchenne muscular dystrophy	1	0.7
Juvenile rheumatoid arthritis	1	0.7
Hydronephrosis	1	0.7
Hypothyroid	1	0.7
Developmental delay	1	0.7
Postinfectious bronchiolitis	1	0.7
None	100	69
Unknown	18	12,4
Total	145^a^	100

*ASD* Atrial Septal Defect, *VSD* Ventricular Septal Defect

^a^One patient had two different co-morbidities

Regarding race and ethnicity, 17 (11.7%) patients belonged to a minority group. Eleven (7.5%) were of Roma origin, 3 (2%) of Afghan origin and 3 (2%) of south East Asian. The remaining patients were Greek.

### Clinical features

Presenting symptoms and clinical findings are shown in Table 2. Almost all patients presented with fever (99.3%) and 112 (77.2%) with acute gastrointestinal symptoms (diarrhea, abdominal pain or vomiting). None of the patients developed clinical ileitis, gut perforation or severe abdominal complications.

**Table 2:** Demographics, clinical and laboratory characteristics and treatment of reported cases (*n* = 145)

	**Total**		**Shock**		**Without shock**		***p*-value**
	*Number*	*Percentage*	*Number*	*Percentage*	*Number*	*Percentage*	
**Total**	145	100	20	13.8	125	86.2	
Age (year), median (IQR)	8.2 (4.2–13.1)		9.5 (6–13.4)			7.8 (3.6–13.1)	0.394
Male	95	65.5	10	50	85	68	0.103
**Clinical manifestations**							
Fever	144	99.3	20	100	124	99.2	0.671
Rash/Mucocutaneous involvement	99	68.3	16	80	83	66.4	0.575
Gastrointestinal symptoms	112	77.2	19	95	93	74.4	0.040
Respiratory symptoms	40	27.6	6	30	34	27.2	0.702
Neurological symptoms	19	13.1	6	30	13	10.4	0.566
**Complications**							
Acute Kidney Injury	23	15.9	10	50	13	10.4	< 0.001
Myocarditis	55	37.9	15	75	40	32	< 0.001
Pericarditis	29	20	3	15	26	20.8	0.461
Coronary Aneurysm	19	13.1	5	25	14	11.2	0.142
Mechanical Ventilation	9	6.2	7	35	2	1.6	< 0.001
ICU admission	33	22.8	17	85	16	12.8	< 0.001
ECMO	1	0.7	1	5	0	0	0.018
**Outcome**							0.019
Death	1	0.7	1	5	0	0	
**Treatment**							
IVIG + Corticosteroid	117	80.6	18	90	99	79.2	0.457
IVIG	134	92.4	20	100	114		0.003
Corticosteroids (2 mg/kg)	117	80.7	17	85	100	80	0.971
Corticosteroids pulse	25	17.2	11	55	14	11.2	< 0.001
Anti-IL1	15	10.3	7	35	8	6.4	< 0.001
Anti-IL6	2	1.4	2	10	0	0	
**Laboratory markes, median (IQR)**							
WBC (× 10^9^/mm^3^)*	14.2 (10–20.5)		17.1 (12.2–23.6)		14.08 (9.6–20)		0.035
Neutrophils (× 10^9^/mm^3^)*	9.7 (6.6–14.5)		14.1 (8.7–20.6)		9.56 (6.5–13.6)		0.017
Lymphocytes (× 10^9^/mm^3^)**	1.13 (0.6–2)		0.45 (0.28–1.12)		1.18 (0.7–2.2)		0.001
Hb (g/dl)**	9.8 (8.8–10.9)		8.8 (8.2–9.8)		9.9 (8.9–11)		0.003
PLT (× 10^9^/mm^3^)**	160 (117–276)		99.5 (66–148)		185 (132–281)		0.003
CRP (mg/l)*	155 (92.5–242.7)		235 (149–350)		152 (89.4–225.3)		0.017
PCT (ng/ml)*	1.69 (0.55–7.56)		8 (2.5–145)		1.56 (0.48–6.98)		0.010
Ferritin (ng/ml)*	535 (277–1144.5)		870 (499–1742.5)		389 (231–1000)		0.006
d-dimers (μg/ml)*	4.31 (2.2–8.4)		6.5 (3.2–11.8)		4.1 (1.82–7.7)		0.185
Troponin (pg/ml)*	11.2 (3–63.5)		55.3 (0.98–148.5)		10.3 (3.5–45.4)		0.891
NT-Pro-BNP (pg/ml)*	1990 (540–5613)		27,197 (2788–38,324)		1834 (465–3716)		< 0.001

*ICU* intensive care unit, *ECMO* extracorporeal membrane oxygenation, *IVIG* intravenous immunoglobulins, *IL* interleukin, *WBC* White Blood Cells, *Hb* Hemoglobin, *PLT* Platelets, *CRP* C-reactive protein, *PCT* procalcitonin, *NT-Pro-BNP* B-type natriuretic peptide

*Maximum value; **Minimum value

Rash or mucocutaneous involvement (bilateral non-purulent conjunctivitis or oral mucocutaneous inflammation signs) were reported in 99 patients (68.3%), respiratory symptoms in 40 (27.6%) and acute kidney injury, indicated by decreased or no urine output or/and elevated serum creatinine, in 23 (15.9%). Regarding cardiac complications, 55 patients (37.9%) developed myocarditis, 29 (18%) had pericarditis, and 19 (13.1%) developed coronary aneurysms (mean z-score: 3.8). Cardiac complications had not fully resolved upon discharge in 11/55 (20%), 1/29 (3.4%) and 5/19 (26.3%) of the aforementioned cases, respectively. Eighteen patients (12.4%) had more than one finding on echocardiography, including 9 (6.2%) with myocarditis plus pericarditis, 5 (3.4%) myocarditis plus coronary involvement, 2 (1.3%) coronary aneurysms plus pericarditis and 2 (1.3%) had all three cardiac complications.

Central nervous system involvement was reported in 19 patients (13%). Neurological symptoms, including headache (8/145), irritability (6/145), lethargy (2/145), photophobia (2/145), hallucinations (1/145) and seizures (1/145) were documented. None of the patients required specific treatment or continued to have symptoms following discharge.

### Laboratory findings

As expected, most patients had significantly elevated inflammatory markers. The most noteworthy laboratory findings are described in Table 2. Median CRP was 155 mg/l (IQR 92.5–242.7), ferritin 535 ng/ml (IQR 277–1144.5), PCT 1.6 ng/ml (IQR 0.55–7.56) and WBC count was 14.2 × 10^9^/mm^3^ (IQR 10–20.5). Almost half of the patients had elevated troponin (60/145, 41.3%; normal value < 14 pg/ml) and/or NT-pro-BNP (72/145, 49.6%; normal value < 300 pg/ml). Lymphopenia and anemia were also noted, with median values of 1.138 × 10^9^/mm^3^ (IQR 0.625–2.015) and 9.8 g/dl (IQR 8.8–10.9) respectively. Blood, urine and/or stool cultures were all negative.

To determine if there is a correlation between variables and disease severity, the need for inotropic support was defined as the most suitable indicator of disease severity. Our data showed that NT-pro-BNP was a significant indicator (*p* < 0.001), with higher values observed in patients with shock (median 27,197 pg/ml, IQR 2788–38,324) compared to those without shock (median 1878 pg/ml, IQR 464–3735). No similar correlation was observed with troponin levels. Additionally, patients with myocarditis (*p* < 0.001, median 4509.5 pg/ml, IQR 1600–14,922) and acute kidney injury (*p* < 0.001, median 8180 pg/ml, IQR 941–32,084) had significantly increased NT-pro-BNP levels compared to patients without myocarditis (median 823.5 pg/ml, IQR 322–2119) and/or without kidney impairment (median 1926 pg/ml, IQR 514–3725.5), respectively. Subsequently, a multivariate logistic analysis was performed (Table 3). Male participants tended to have 66% lower risk of developing shock compared to females (OR 0.34, 95% CI 0.09, 1.2, *p* = 0.09). Furthermore, patients with myocarditis had a 3.64 times higher risk of developing shock compared to patients without myocarditis (95% CI 0.97, 13.7, *p* = 0.055). Acute kidney injury and corticosteroid pulses were found to be determining factors of disease severity. The development of acute kidney injury was associated with an 8.5 times higher risk (95% Cl 2.1, 34.9, *p* = 0.003) of developing shock, and corticosteroid pulse was associated with a 9.6 times higher risk (OR 9.6, 95% CI 2.4, 37.6).

**Table 3:** Multivariate logistic analysis

	**Odds ratio**	**95% Confidence interval**		***P* values**
**Sex male**				
**Yes**	0.336	0.095	1.191	0.091
**Age**	0.967	0.851	1.099	0.617
**Comorbidities**				
**Yes**	0.436	0.083	2.275	0.325
**Myocarditis**				
**Yes**	3.649	0.972	13.695	0.055
**Respiratory symptoms**				
**Yes**	0.929	0.219	3.930	0.921
**Acute Kidney Injury**				
**Yes**	8.537	2.088	34.905	0.003
**Steroid Pulses**				
**Yes**	9.630	2.463	37.647	0.001

Most of the cases had serological evidence of SARS-CoV-2 infection (112/145, 77%), and 21 (14.5%) had a positive RT-PCR test for SARS-CoV-2.

### Treatment

In accordance with international guidelines, management included antibiotics (until bacterial causes were excluded), immunomodulatory therapy and antiplatelet or anticoagulation medication, as well as supportive treatment [14–17]. Most of the patients (80.6%) received both intravenous immunoglobulin (IVIG) at a dose of 2 g/kg in all centers and corticosteroid treatment (2 mg/kg or pulses). IVIG alone was used in 13/145 patients (8.9%), while corticosteroids were given as monotherapy in 2/145 (1.3%). Regarding the adjunct use of biologics for refractory cases, 10.3% of the patients in this cohort were also treated with anakinra (15/145), while tocilizumab was administered in 2 cases (1.3%) (Table 2). The administration of anakinra treatment commenced at an average of 2.7 days of hospitalization (IQR 1–3.5). The median interval from symptom onset to hospital admission was 5.9 days (IQR 3–6) and the median period from hospital admission to diagnosis and initiation of treatment was 1.2 days (IQR 0–2). Finally, the median time from symptom onset to initiation of treatment was 7 days (IQR 4–7).

Thirty-three patients (22.8%) were admitted to the PICU, among which 20 (13.8%) presented with shock and needed inotropic support. Nine patients (6.2%) required mechanical ventilation and 1 received ECMO. The mortality rate was 0.68% (1/145).

The incidence of MIS-C was estimated at 0.69/1000 SARS-CoV-2 infections, based on the national registry of SARS-CoV-2 infections maintained by the National Public Health Organization.^2^

## Discussion

This national, collaborative, retrospective, descriptive study includes all patients hospitalized with MIS-C from the beginning of the COVID-19 pandemic until the end of the second year. Demographics, clinical and laboratory characteristics, treatments and outcomes are described. There was a wide spectrum of signs, symptoms, and disease severity. However, with a high index of suspicion and timely initiation of treatment, most patients had a positive outcome.

A more favorable disease course was observed in this study compared to others. Thirty-three patients (22.8%) were admitted to the PICU, 13.8% received inotrope support, 6.2% mechanical ventilation, only one patient required ECMO, and one died. Whittaker et al., in a case series of 58 patients, reported 50% of the recorded cases were admitted to the PICU, 47% received inotropes and 43% were placed on mechanical ventilation [18]. Dufort et al., in surveillance conducted in New York, including 99 cases of MIS-C, reported that up to 80% of the patients were admitted to the PICU, of which 62% received vasopressor support, 10% required mechanical ventilation, and 1 child ECMO; the mortality rate was 2% [19]. Feldstein et al., conducted a study with 186 MIS-C patients from 26 states of the USA [12]. Most of those who were admitted to the PICU (80%), 20% required mechanical ventilation, 48% vasoactive support and 4 died. In a multicenter study from Spain, that included 152 patients with MIS-C, the findings indicated that 53.3% presented with shock and 44.7% were admitted to the PICU. There were no fatalities [20]. In the current study, most of the patients received therapy with little delay (mean time 1.2 days) since there was a high index of suspicion of this clinical entity. Early diagnosis and treatment may account for the milder course in the current cohort compared to patients in earlier studies.

Interestingly, in MIS-C the cardiovascular system seems to be the most severely affected. The underlying mechanisms leading to myocardial dysfunction in MIS-C have not been yet fully elucidated [21–23]. Based on recent reports, the main cardiac abnormalities found in patients who manifest MIS-C are left ventricular dysfunction, coronary artery dilatation or aneurysms, myocarditis, elevated cardiac enzymes (troponin or NT-pro-BNP), and/or pericarditis [21, 22, 24, 25]. Following appropriate immunomodulatory treatment, the above-mentioned manifestations seem to improve [26]. Similarly, in the current cohort the cardiac complications resolved in most patients before discharge. In keeping with international guidelines, close follow-up for potential long-term complications is required.

With regards to cardiac biochemical markers, recent evidence suggests a direct link between NT-pro-BNP levels and severe MIS-C [27, 28]. However, no relationship was observed between severe MIS-C and troponin levels [27]. These observations were confirmed in this cohort. The results probably indicate that NT-pro-BNP levels may provide a better prediction and may be a monitoring tool for the disease course.

Multivariate logistic analyses showed that acute kidney injury and myocarditis were the most important variables that increased the risk of developing shock. Moreover, as expected the administration of corticosteroid pulses proved to be a determining factor, as well. Corticosteroid pulses were given to patients with disease refractory to the initial treatment or as first-line treatment to patients with cardiac involvement. As patients with more severe disease received corticosteroid pulses, the correlation between disease severity and administration of corticosteroid pulses was foreseeable.

With regards to treatment offered to patients with MIS-C, given the lack of a randomized controlled trial the most effective strategy is still under discussion. The BATS study concluded that the outcomes of patients who received IVIG plus corticosteroids or monotherapy with IVIG or with corticosteroids were comparable [29, 30]. Other studies suggested that the combination of IVIG plus corticosteroids is more efficacious [31, 32]. Similarly, in this study, 80.6% of patients received both IVIG and corticosteroid treatment, with favorable outcomes. Patients with refractory disease or cardiac impairment also received second-line treatment with biologics (anakinra or tocilizumab) according to the recommendations of the American College of Rheumatology and the National Institute of Health [14, 15]. Taddio et al. found that the use of anakinra within the first 48 h was linked to a reduced likelihood of persistent heart disease [33]. In this cohort we could also confirm that early introduction of anakinra was associated with favorable cardiac outcome in the few patients who received this treatment. Our experience suggests that early recognition of this novel disease and immediate initiation of treatment contributed to positive outcomes.

The incidence of MIS-C observed in this cohort was significantly higher than that described in the CDC report (1 per 1449 SARS-CoV-2 infections vs. 1 per 3000–4000 SARS-CoV-2 infections) [34]. A multicenter study conducted in Spain also exhibited a lower incidence rate compared to this study (1 per 3700 SARS-CoV-2 infections) [20]. On the other hand, Germany recorded an estimated incidence rate of 1 per 1357 SARS-CoV-2 infections in 2021 [35]. One explanation for these differences may be that in the first two waves of the pandemic, compared to the third wave, SARS-CoV-2 testing in schoolchildren in Greece was limited. Asymptomatic children or young patients with minor illness during that period might not have been detected and hence, not enrolled in the national registry. It is worth noting that this study was conducted during a period when the Alpha and Delta variants of the SARS-CoV-2 were primary. However, milder cases may have not been diagnosed. Finally, some studies detected a significant difference in the incidence among various groups, mainly focusing on race and/or ethnicity [36, 37]. In the Greek population, these differences were not detected. Moreover a recent report noted a decreasing incidence of MIS-C in Greece during the first three successive pandemic waves [38]. Considering all this information, further studies are required to estimate the accurate incidence of MIS-C amongst other ethnicities and countries.

A major question is to what extent the vaccination against SARS-CoV-2 protected children from developing MIS-C. In this cohort, immunization history was not recorded in all cases. However, vaccination of schoolchildren was limited during the study period, as vaccination of children 5–11 years of age started in December 2021. One case of a fully vaccinated adolescent who developed MIS-C was noted. We did not observe any cases of MIS-C following vaccination (MIS-V). Recent studies underlined the possible protective effect of SARS-CoV-2 vaccination against MIS-C by finding a lower incidence of MIS-C cases post-vaccination [39–41]. A study conducted in the USA, included 102 patients ages 12–18 years with MIS-C and found an estimated effectiveness of 2 doses of the Pfizer-BioNTech vaccine against MIS-C of 91% (95% Cl = 78–97%) [39]. Another study performed in France that included 33 adolescents with MIS-C, described an efficacy of the Pfizer-BioNTech and Moderna vaccines of 95% [40]. Both studies shared some limitations. On the other hand, a few case reports described fully immunized patients who developed MIS-C despite previous vaccination [42–45]. Moreover, a handful of studies reported MIS-C cases as an adverse effect of vaccination (MIS-V) [46–50]. Nonetheless, there is no clear evidence to support the notion that vaccination against COVID-19 may be a trigger for MIS-C. However, even if a correlation with vaccination exists, the frequency of patients with MIS-V does not exceed one case per million vaccinations [51].

MIS-C is a novel and challenging disease entity that we still do not completely comprehend. Current literature suggests that it is not the result of acute viral infection but a post-infectious phenomenon that has been directly or indirectly linked to causing a massive release of pro-inflammatory cytokines and immune dysregulation [52–57], categorizing MIS-C as a hyper-inflammatory syndrome and/or cytokine release (storm) syndrome [57, 58]. However, even with these insights, definite conclusions cannot be drawn regarding the underlying immune pathogenesis of this complex, hyper-inflammatory disease.

### Limitations

The current study had certain limitations. Firstly, data were collected retrospectively. Upon appearance, the case definition and the most appropriate treatment of this new syndrome were not clearly defined. As a result, investigation and management in each center were based on knowledge and experience. Secondly, detection of asymptomatic SARS-CoV-2 infection was limited during the first two waves of the pandemic. Consequently, some cases with mild or no symptoms were not registered, which affected the denominator in the incidence estimation, as previously mentioned.

## Conclusions

MIS-C is a novel, appreciatively infrequent; yet serious disease entity. The most frequent cardiac manifestations found in this study included myocarditis and pericarditis, which resolved in most patients prior to discharge. Acute kidney injury and myocarditis were associated with higher risk of developing shock. Moreover, NT-pro-BNP levels were found to be a possibly reliable indicator that can enhance the prediction and monitoring of the disease course. Immediate initiation of combined immunomodulatory therapy (IVIG and corticosteroids) was shown to be effective and the mortality rate remained low. However, the exact rate of residual cardiac involvement and chronic complications remains to be clarified. Further research is required to illuminate the pathogenesis, risk factors and optimal management of MIS-C.

## Data Availability

The datasets utilized and assessed in this study can be obtained from the corresponding author upon a reasonable request.

## Abbreviations

Anti-IL1: Anti-interleukin 1
BATS: Best Available Treatment Study
BMI: Body mass index
CDC: Centre for Disease Control and Prevention
COVID-19: Coronavirus disease 2019
CRP: C-reactive protein
ECMO: Extracorporeal membrane oxygenation
GDPR: General Data Protection Regulation
IQR: Interquartile range
IVIG: Intravenous immunoglobulin
MIS-C: Multisystem inflammatory syndrome in children
MIS-V: MIS-C following vaccination
NT-Pro-BNP: N-terminal pro- B-type natriuretic peptide
PCT: Procalcitonin
PICU: Pediatric intensive care unit
PIM-TS: Pediatric inflammatory syndrome temporarily associated with Sars-CoV-2
RT-PCR: Reverse-transcriptase protein chain reaction
SARS-COV2: Severe acute respiratory syndrome coronavirus 2
WBC: White blood cells
WHO: World Health Organization

## Footnotes

1 The pediatric clinics of the Hippokration General Hospital the First and Third Department of Paediatrics of the Aristotle University of Thessaloniki, ‘Aghia Sophia’ Children's Hospital the first Department of Paediatrics of the National and Kapodistrian University of Athens, Papageorgiou General Hospital the fourth Department of Pediatrics of the Aristotle University of Thessaloniki, Genimatas General Hospital of Thessaloniki, Department of Paediatrics in the Faculty of Medicine of the School of Health Sciences of the University of Thessaly, Department of Paediatrics of the Medical School of the University of Crete, Department of Paediatrics of Patras Medical School of University General Hospital of Patras, Penteli Children's Hospital, General University Hospital “Attikon” the Third Department of Paediatrics, of the National and Kapodistrian University of Athens, Metropolitan Hospital in Athens and IASO Children’s Hospital in Athens.

2 https://eody.gov.gr/en
